# Polarization-Dependent Quasi-Far-Field Superfocusing Strategy of Nanoring-Based Plasmonic Lenses

**DOI:** 10.1186/s11671-017-2154-1

**Published:** 2017-06-02

**Authors:** Hao Sun, Yechuan Zhu, Bo Gao, Ping Wang, Yiting Yu

**Affiliations:** 1 Key Laboratory of Micro/Nano Systems for Aerospace, Ministry of Education, Northwestern Polytechnical University, Xi’an, 710072 China; 2 Key Laboratory of Micro- and Nano-Electro-Mechanical Systems of Shaanxi Province, Northwestern Polytechnical University, Xi’an, 710072 China

**Keywords:** Polarization, Nanoring-based plasmonic lenses, Subwavelength structures, Superfocusing, Geometric optics

## Abstract

The two-dimensional superfocusing of nanoring-based plasmonic lenses (NRPLs) beyond the diffraction limit in the far-field region remains a great challenge at optical wavelengths. In this paper, in addition to the modulation of structural parameters, we investigated the polarization-dependent focusing performance of a NRPL employing the finite-difference time-domain (FDTD) method. By utilizing the state of polarization (SOP) of incident light, we successfully realize the elliptical-, donut-, and circular-shape foci. The minimum full widths at half maximum (FWHMs) of these foci are ~0.32, ~0.34, and ~0.42 *λ*_0_ in the total electric field, respectively, and the depth of focus (DOF) lies in 1.41~1.77 *λ*_0_. These sub-diffraction-limit foci are well controlled in the quasi-far-field region. The underlying physical mechanism on the focal shift and an effective way to control the focusing position are proposed. Furthermore, in the case of a high numerical aperture, the longitudinal component, which occupies over 80% of the electric-field energy, decides the focusing patterns of the foci. The achieved sub-diffraction-limit focusing can be widely used for many engineering applications, including the super-resolution imaging, particle acceleration, quantum optical information processing, and optical data storage.

## Background

Along with the development of super-resolution imaging [1], particle acceleration [2], quantum optical information processing [3], and polarization-dependent optical data storage [4], surface plasmonic devices are widely applied in these regions by modulating the plasmon resonance in a subwavelength magnitude. The plasmonic lens (PL), as a typical device, possesses the perfect imaging capability which was firstly proposed by J. B. Pendry in 2000 [5], and the sub-diffraction-limit optical imaging was experimentally demonstrated by X. Zhang et al. 5 years later [6]. However, the imaging plane was confined to the extremely near field as the evanescent waves decrease exponentially, indicating a divergent optical field. This limitation makes it impracticable for standard optical microscopes [7].

In recent years, several nanostructure-based PLs have been investigated [8–24]. These PLs can not only realize the plasmonic focusing in plane [8] or in the near field [9] but can also possess the focusing ability in the far field [10–13] which were generally imaged by the scanning optical microscope [14]. However, these PLs showed a great difficulty to realize the focusing beyond the diffraction limit, until the dispersion relation of the metal-insulator-metal (MIM) waveguide was employed for the nanoslit-based PLs to modulate the phase at the subwavelength scale [11, 15–17]. The precise phase modulation contributes to the sub-diffraction-limit focal line, and the linearly polarized light is generally applied as the incident light for these lenses. But, by simply extending the similar design method of one-dimensional nanoslit lenses into two dimensions, the circular-shape focus cannot be realized when the rotationally symmetric PL was illuminated by the linearly polarized light [18, 19], indicating the strongly polarization dependence of the focusing performance. Furthermore, the focal length was seriously deviated from the numerical calculation according to the wavefront reconstruction theory, especially for the nanoring-based PLs [18].

The nanoring-based PLs with the rotational symmetry, which are named as nanoring-based plasmonic lenses (NRPLs) for simplicity in the following discussion, are recognized as the substitution for the conversional refractive lenses in the subwavelength focusing systems. But from the perspective of the excitation of surface plasmon polaritons (SPPs), the linearly polarized light is unsuitable for the NRPLs as the excitation efficiency is proportional to the radial electric-field component of the incident light. Comparatively, the radially polarized light with the cylindrical symmetry in polarization adapts to the structural property of NRPLs [25]. Additionally, utilizing a donut-shape aperture stop [26, 27] or Fresnel zone plate [28], this polarized light has been applied to realize the subwavelength circular-shape focus. Thus, the radially polarized light was generally applied as the incident light of NRPLs [20–23]. Compared with the subwavelength focusing with the spatial filter, these PLs possess the ability to modulate the phase of incident light in the subwavelength waveguide. The excitation of surface plasmonic (SP) waves can enhance the transmission of electromagnetic waves. However, the superfocusing capability of plasmonic lens in the far field has not been demonstrated. Furthermore, although the composite NRPL has been proposed to modulate the focal length [24], the focal length failed to be effectively controlled and the electric-field energy is still concentrated in the center of the end surface of the lens.

In this paper, we present the theoretical design and numerical study of a NRPL, with the emphasis on the realization of superfocusing by utilizing the polarization property of incident light. We describe the theoretical design of NRPLs in our research and provide the focusing performance in the output region based on the finite-difference time-domain (FDTD) numerical simulation. To investigate the polarization dependence of the focusing performance, the linearly, circularly, azimuthally and radially polarized lights are all considered for illumination. We discuss the electric-field distribution features in the output region, including the sub-diffraction-limit focusing, the shapes of foci, and the modulation of focal length, and point out the importance of the coaxial condition on the superfocusing performance.

## Methods

The NRPLs investigated in this work were designed by using the wavefront reconstruction theory which was widely applied to the nanoslit-based PLs [11, 15]. In order to achieve the focus at the desired position, the relative phase delay caused when the light passes through the *i*-th individual nanoring needs to satisfy the following condition based on the geometric optics:

1 $$ -\varDelta \phi \left({r}_i\right)=\frac{2\pi \sqrt{f_0^2+{r}_i^2}}{\lambda_0}-\frac{2\pi \sqrt{f_0^2+{r}_1^2}}{\lambda_0}+2 n\pi $$

where −Δ*ϕ*(*r*_*i*_) is the relative phase difference between the first nanoring in the inner center and the *i*-th nanoring, *r* is the radius, *λ*_0_ is the free-space wavelength of the incident light, *n* is an arbitrary integer, and *f*_0_ is the designed focal length.

**Fig. 1:**
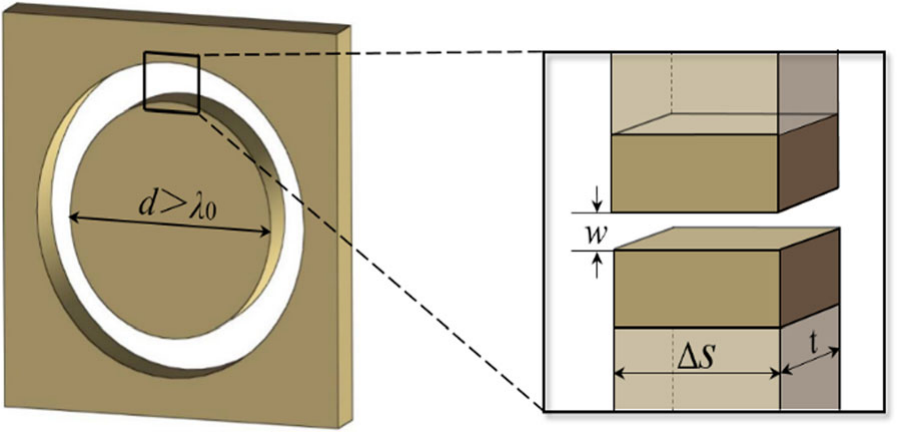
The MIM waveguide model of an individual nanoring embedded in the gold film. The *inset* gives an enlarged view of an arbitrary small portion of the nanoring

The basic comprising element of the surveyed NRPLs is the nanorings patterned in the metallic film. According to Ref. [29], when the diameter is larger than the wavelength of incident light, the air nanoring surrounded by metallic walls can be approximated to the MIM waveguide model as the inset illustrates in Fig. 1. The phase delay is primarily defined by the real part of the propagation constant *β*, expressed as Re(*β*)•*t*, where *t* is the thickness of the nanoring. Based on the dispersion relationship, the complex propagation constant *β* can be calculated as:

2 $$ \tanh \left(\frac{w\sqrt{\beta^2-{k}_0^2{\varepsilon}_d}}{2}\right)=-\frac{\varepsilon_d\sqrt{\beta^2-{k}_0^2{\varepsilon}_m}}{\varepsilon_m\sqrt{\beta^2-{k}_0^2{\varepsilon}_d}} $$

where *k*_0_ represents the wave vector in vacuum and *ε*_*d*_ and *ε*_*m*_ are the permittivity of dielectric and metal, respectively. Base on Eq. (2), we can see that the propagation constant *β* is dependent on the width of a nanoring. Thus, for the flat lens with a given thickness *t*, the caused phase delay is simply determined by the width *w* of the individual nanoring when the light passes through it. In our research, the dielectric is set to be air with the permittivity *ε*_*d*_ = 1, and the gold film with a thickness *t* of 400 nm is employed, whose permittivity at the incident wavelength of 650 nm is *ε*_*m*_ = −12.8915 + 1.2044i [15]. Additionally, as we previously reported [16], the coupling effect of the propagating lights in the two adjacent MIM waveguides also plays an important role on the phase delay, especially when the spacing metallic wall is smaller than twice the skin depth *δ*_*m*_, which can be estimated by [30]:

3 $$ {\delta}_m=\frac{1}{k_0}{\left|\frac{\mathrm{Re}\left({\varepsilon}_m\right)+{\varepsilon}_d}{\mathrm{Re}{\left({\varepsilon}_m\right)}^2}\right|}^{\frac{1}{2}} $$

Accordingly, the calculated skin depth *δ*_*m*_ is about 28 nm. By considering the coupling effect, a nanoslit-based PL with the superfocusing capability of 0.38 *λ*_0_ in resolution was reported in our previous research [16]. Here, to prominently analyze the influence of the state of polarization (SOP) on the focusing performance, the spacing walls between two adjacent nanorings are designed to be 100 nm, much larger than 2*δ*_*m*_ to eliminate the coupling effect.

**Fig. 2:**
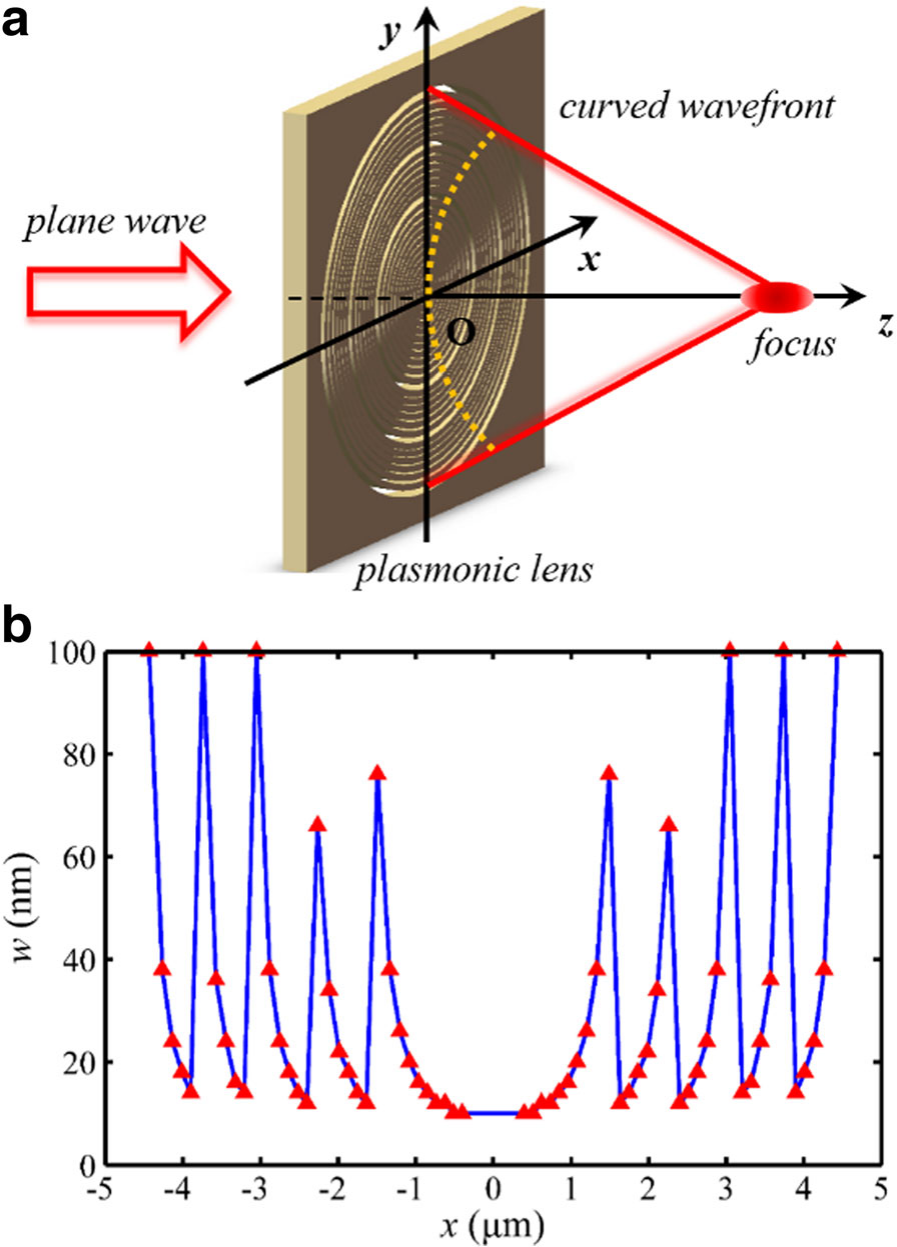
Schematic of the NRPL. **a** The incident light normally illuminates the lens. **b** Structural parameters of the lens. The coordinate values of the *red triangles* represent the radius *r* and width *w* of the corresponding nanoring

The schematic of the designed NRPL is shown in Fig. 2, and a total number of 32 concentric nanorings are included to reconstruct the wavefront. The width of the nanoring for the desired phase modulation range from 10 to 100 nm. The aimed focal length *f*_0_ is 1300 nm (2 *λ*_0_). To efficiently utilize the MIM waveguide model, the minimum diameter of the innermost nanoring is set to be 800 nm. Furthermore, to avoid the focal shift as discussed in our previous work [15], the total phase difference is as large as 10π, with a predicted numerical aperture (NA) of 0.96. As a result, the theoretical Rayleigh diffraction limit, calculated by 0.61 *λ*_0_/NA [31], is 413 nm (~0.64 *λ*_0_).

To investigate the SOP influences of the incident light on the spatial intensity distributions, especially the focusing performance, the designed NRPL was illuminated by the linearly, circularly, azimuthally, and radially polarized light, respectively. All the cases were computed by the FDTD numerical simulations. According to the matrix optics, the different polarized lights can be described by the Jones matrix formalisms, and the corresponding matrix expression was applied to define the incident light. The boundary of the model was perfectly matched layer (PML) with a layer number of 12. To balance the computational accuracy and the memory consumption in the simulations, the mesh size was set to be 10 nm in the output region and 5 nm around the focal region.

## Results

### I Linear Polarization

**Fig. 3:**
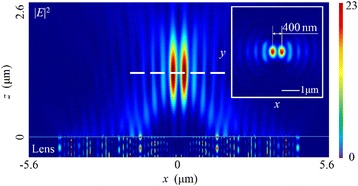
Intensity distribution pattern of the total electric field |*E*|^2^ in the case of linearly polarized incident light. The *inset* shows the intensity pattern in the focal plane. The focal length is 1215 nm (a deviation of 6.54%). The FWHMs of both foci are ~0.32 *λ*_0_, with 400 nm apart from each other, and the depth of focus (DOF) is ~1.68 *λ*_0_

For the linearly polarized light, the SOP is spatially homogeneous, and in this case, the direction of the electric vector is parallel to the *x* axis. When the light illuminates the NRPL, there exist two foci, distributing 400 nm away from each other in the total electric field |*E*|^2^ as presented in Fig. 3. Though the full widths at half maximum (FWHMs) of both are 210 nm (~0.32 *λ*_0_) in the focal plane, the simulation result indicates that the intensity distribution is apparently different from the design based on the wavefront reconstruction theory where there should be a circular-type focus exactly on the *z* axis (also called the optical axis).

**Fig. 4:**
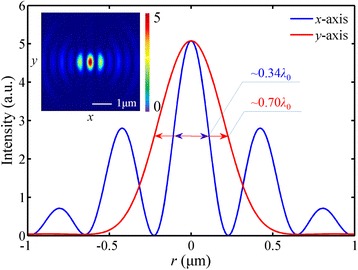
Intensity distribution of the transverse component |*E*_*r*_|^2^ in the focal plane. The *inset* shows the elliptical-shape focus. The focal length is 1425 nm (a deviation of 9.62%). |*E*_*r*_|^2^ occupies 20.2% of the total electric energy. The DOF is ~1.41 *λ*_0_

To analyze the differences between the simulation and theoretical design, the intensity distributions of the electric-field components are investigated. As presented in Fig. 4, there appears an elliptical-shape focus and the FWHM in *x-* and *y-*direction is 220 nm (~0.34 *λ*_0_) and 457 nm (~0.70 *λ*_0_), respectively. This pattern agrees well with the experimental results of Ref. [18] where the same polarized light was applied. However, the simulation shows that the distribution pattern of |*E*|^2^ is similar to the pattern of the longitudinal component |*E*_*z*_|^2^ which occupies 79.8% of the total electric energy. Therefore, the difference is mainly attributed to the extraordinary distribution of |*E*_*z*_|^2^.

**Fig. 5:**
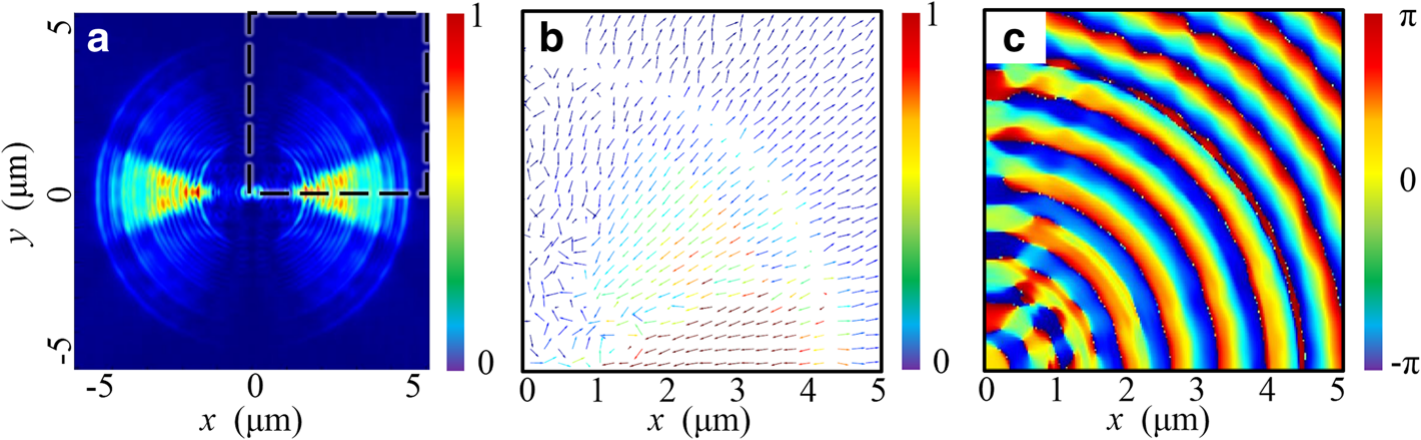
The electric-field distribution properties in the cross section just 50 nm above the end surface of the lens. **a** Normalized intensity distribution of |*E*|^2^. **b** The Poynting-vector distribution within the *dashed-line* region in **a. c** The corresponding phase distribution of *E*_*z*_

This phenomenon can be ultimately explained by the transmission property of the NRPL. On the one hand, the excitation of SPPs at the interfaces of metal and dielectric generally depends on the local polarization direction of the incident light. The transverse electric (TE) waves cannot contribute to the excitation. On the other hand, due to the subwavelength structure of the MIM waveguide, only the SP waves can propagate through this lens [32]. With the rotational symmetry of the lens, the local transverse magnetic (TM) component changes with the azimuthal angle *θ* in the cosinoidal form. Therefore, as shown in Fig. 5a, the intensity distribution of |*E*|^2^, which is just above the end surface of the lens, is concentrated in the near *y* = 0 region (−π/4 < *θ* < π/4). Correspondingly, the Poynting vectors propagate along the radial direction on the end surface, as presented in Fig. 5b. Thus, the vector direction of *E* is basically parallel to the optical axis, which forms the main content of *E*_*z*_. Due to the symmetrically constructive interference, there appear two foci in the focal plane instead of a circular-type focus.

### II Circular Polarization

**Fig. 6:**
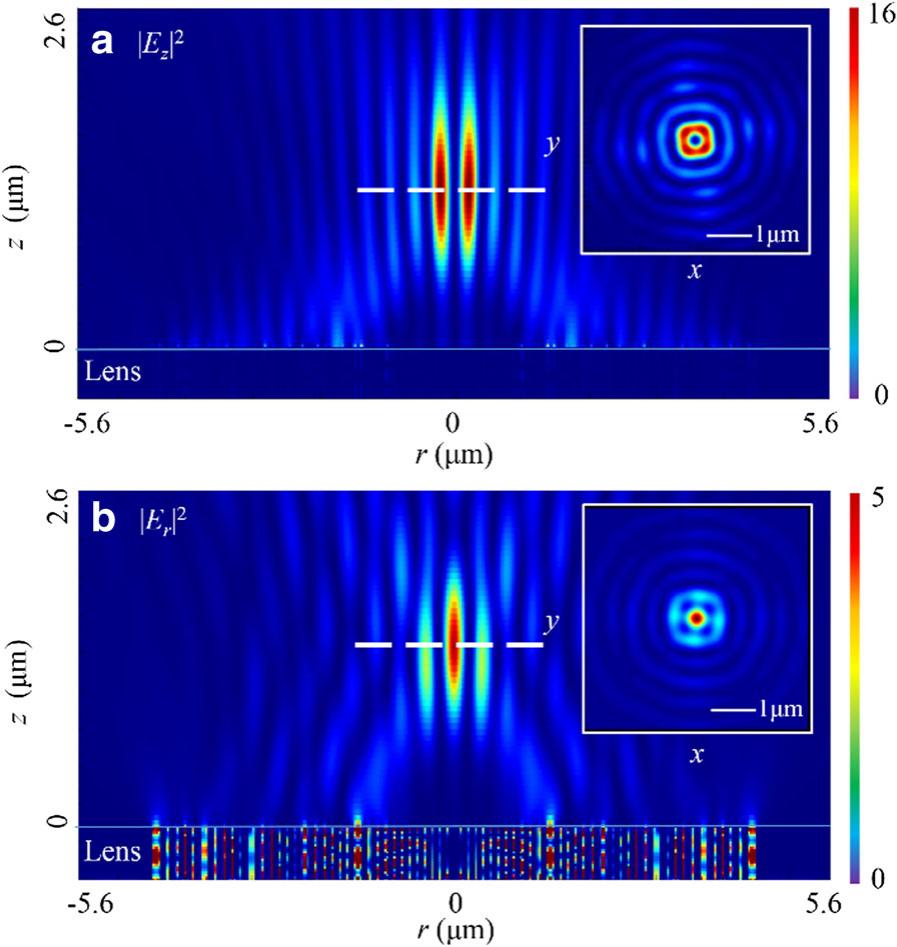
Intensity distribution patterns of |*E*_*z*_|^2^ and |*E*_*r*_|^2^ in *r-z* plane in the case of circularly polarized light. **a** In |*E*_*z*_|^2^, the FWHM, DOF, and focal length is ~0.32 *λ*_0_, ~1.65 *λ*_0_, and 1185 nm, respectively. **b** In |*E*_*r*_|^2^, the FWHM, DOF, and focal length is ~0.45 *λ*_0_, ~1.68 *λ*_0_, and 1405 nm, respectively

As the state of circularly polarized light changes with the time periodically, the simulated results are the time-averaged field distribution. When the lens is illuminated by this polarized light, there forms a donut-shape focus in |*E*_*z*_|^2^. As shown in Fig. 6a, the focal length in this field is 1185 nm, showing a 8.85% deviation to the designed value. The width of the donut is 210 nm (~0.32 *λ*_0_), and the radius is 400 nm. The depth of focus (DOF) is ~1.65 *λ*_0_. The weight of |*E*_*z*_|^2^ is 80.6% of the total electric energy. Additionally, in |*E*_*r*_|^2^, the superposition in the spatial domain generates a circular focus with the 1405-nm focal length (a deviation of 8.08%). The FWHM is 295 nm (~0.45 *λ*_0_) in this field, and the DOF is ~1.68 *λ*_0_. Further, both distribution patterns in *r-z* plane resemble those in the *x*-*z* plane in the case of linearly polarized incident light. By taking into account the radial electric-field component, the FWHM can be reduced to 222 nm (~0.34 *λ*_0_).

### III Azimuthal Polarization

**Fig. 7:**
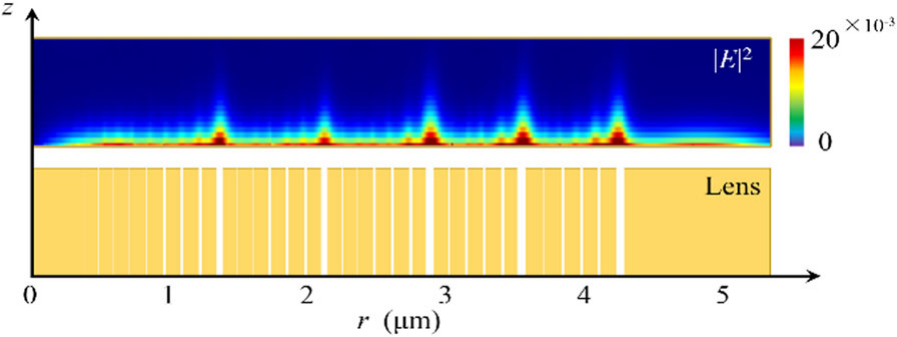
Intensity distribution pattern in the lens region and its cross-sectional view. The transmission distance of the non-SP waves in nanorings is proportional to the slit width

For the azimuthally polarized incident light, the electric vectors are perpendicular to the radial direction, which are parallel to the gold/vacuum interface of the NRPL. As the azimuthally polarized light illuminates the lens, the local TE waves fail to excite the SPPs on the interface. Thus, the transmission distance in nanorings is proportional to their widths as presented in Fig. 7. Since both the structure and illumination are of rotational symmetry, only half of the intensity distribution pattern and the structure of the NRPL are shown. The transmitted light can be neglected, and there is no distinct focus in the output region.

### IV Radial Polarization

**Fig. 8:**
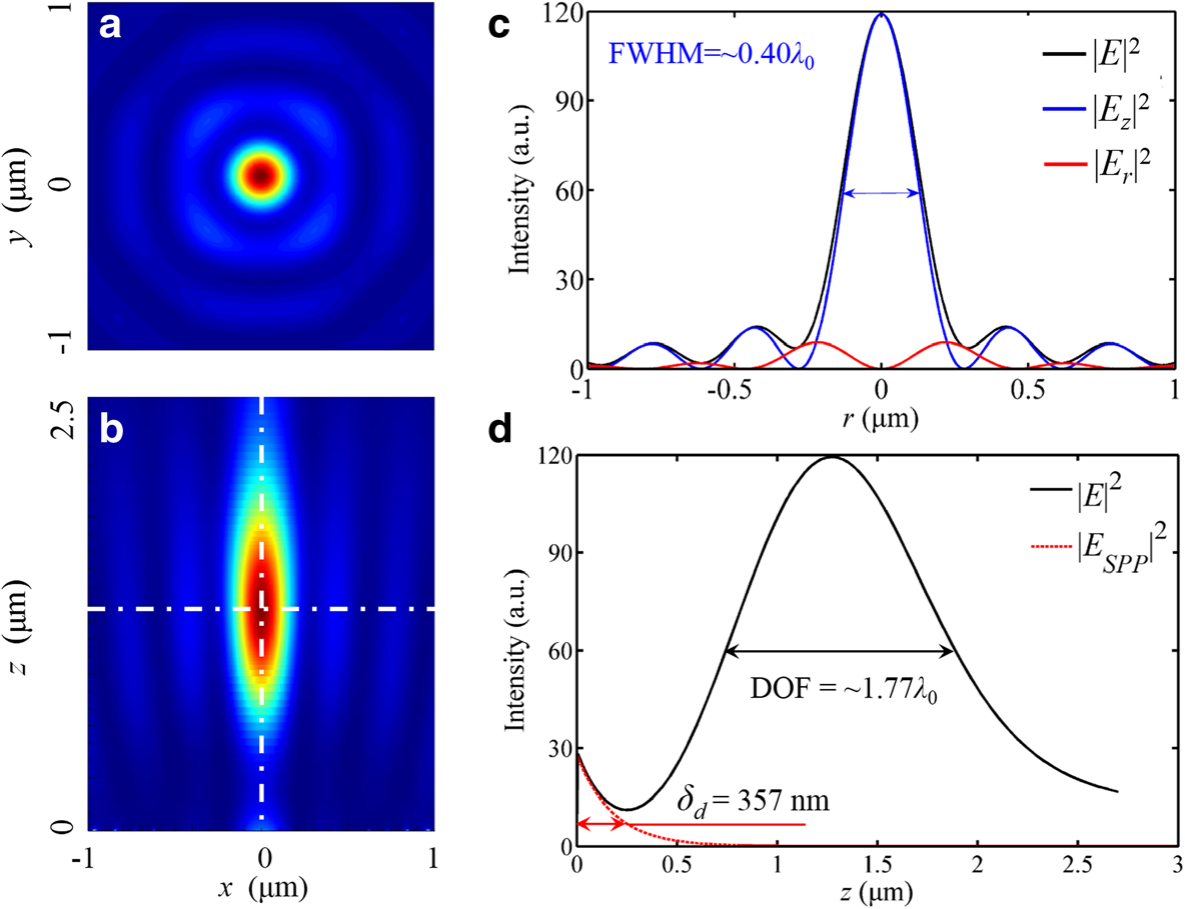
Intensity distribution of |*E*|^2^ in the case of radially polarized incident light. **a**, **b** The distribution in the focal plane and that in *x*-*z* cross section. **c** The intensity profile in the radial direction. **d** The intensity profile along the optical axis where the *black solid line* is the total electric field intensity distribution in simulation and the *red dashed line* is the calculated curve of SPP. The focal length is 1275 nm (a deviation of 1.92%). The FWHM of |*E*|^2^ and |*E*_*z*_|^2^ is 272 nm (~0.42 *λ*_0_) and 260 nm (~0.40 *λ*_0_), respectively. The DOF is ~1.77 *λ*_0_

Corresponding to the azimuthally polarized light, the radially polarized light can be considered as the local TM wave, and this polarization property matches to the excitation condition of SPPs, which contributes to a higher maximum intensity at the focus. In total electric field *E*, the maximum intensity is five times larger than that for the linearly polarized incident light. Additionally, there is a circular-shape focus with the 276-nm (~0.42 *λ*_0_) FWHM in |*E*|^2^, as shown in Fig. 8. The simulated intensity distribution is much similar to the focusing capability of the high NA refractive lens [33]. Furthermore, this focusing performance is still dependent on |*E*_*z*_|^2^, which occupies 82.0% of the total electric energy.

**Fig. 9:**
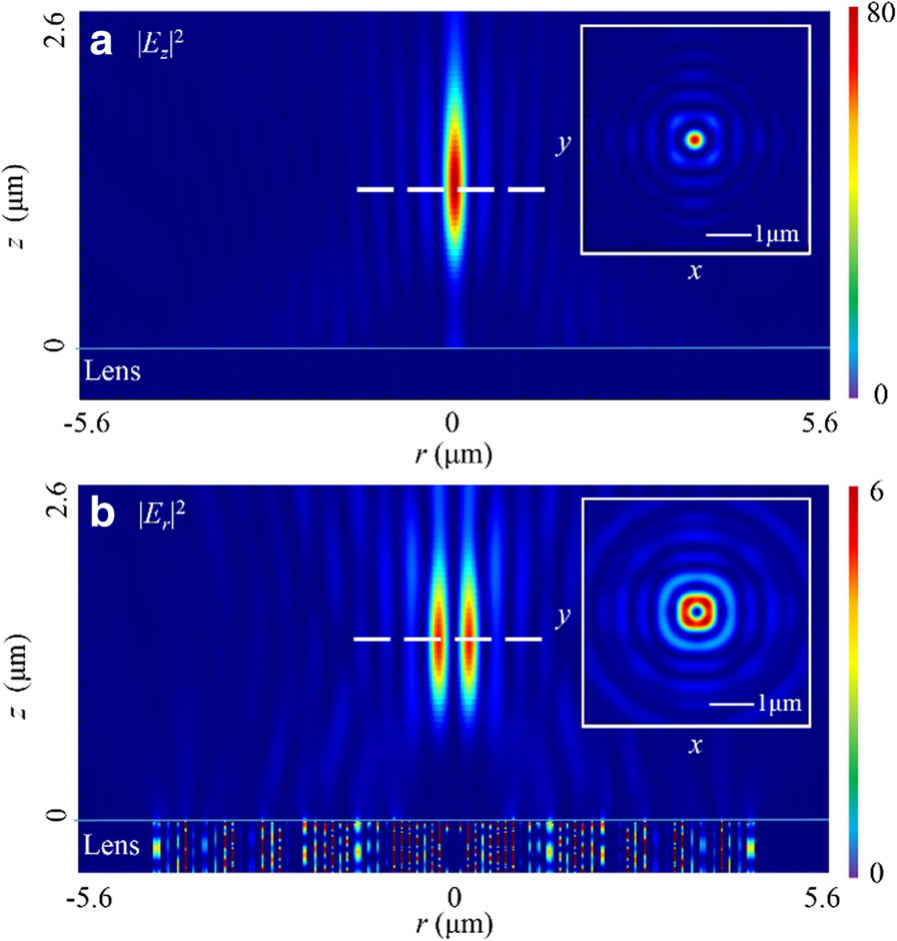
Intensity distribution patterns of |*E*_*z*_|^2^ and |*E*_*r*_|^2^ in *r-z* plane in the case of radially polarized light. **a** |*E*_*z*_|^2^ pattern in the *r-z* plane. The *inset* shows a circular-shape focus in the focal plane. **b** The transverse |*E*_*r*_|^2^ pattern in the *r-z* plane. The *inset* shows a donut-shape focus in the focal plane

Different from the former cases, there is a circular-shape focus in |*E*_*z*_|^2^. Besides, this component also determines the distribution pattern in |*E*|^2^. As presented in Fig. 9a, the FWHM in |*E*_*z*_|^2^ is 260 nm (~0.40 *λ*_0_) which is close to that of the focal line in the case of nanoslit-based PL [16]. Particularly, the focal length is 1275 nm. Compared with the designed value, the relative error decreases to 1.9%. However, the focal length is 1455 nm (a deviation of 11.2%) in |*E*_*r*_|^2^. As presented in Fig. 9b, there is a donut-shape focus with the 227-nm width (~0.35 *λ*_0_) in this field. The DOF is ~1.60 *λ*_0_.

**Fig. 10:**
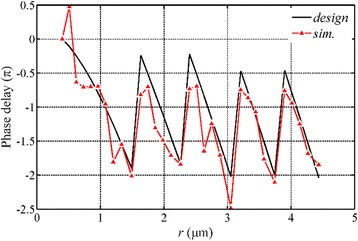
Phase analysis of the NRPL under the radially polarized incident light

The phase delay of the SP waves in nanorings is investigated, as presented in Fig. 10. The simulation indicates that the phase modulation is dramatically influenced by the structural parameters of the NRPL and the simulated phase delays between the incident surface and the output surface are basically identical to the calculated values based on Eq. (2). On the end surface of the lens, the SP waves still propagate along the radial direction and there is a hot spot at the center of the surface, whose intensity is one fifth of the focus intensity. The constructive interference of the SP waves, with the rotational symmetric distribution, constructs the propagating waves and realizes the circular-shape focus in the quasi-far field.

## Discussions

### I Superfocusing Capability of NRPLs

As the incident lights with different SOPs are applied, including the linear, circular, and radial polarizations, the sub-diffraction-limit foci can be realized. Although the focus shape is influenced by the SOP, the characteristic sizes of these foci all overcome the Rayleigh diffraction limit (413 nm). The simulation results successfully demonstrate the superfocusing capability of the NRPL, and the intensity distribution in the focal plane is similar to the Bessel function which is used to describe the non-diffraction beam.

**Fig. 11:**
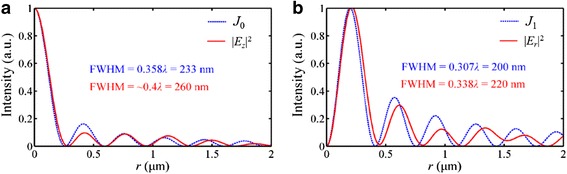
Intensity profiles of the NRPL in the focal plane under the radially polarized incident light. **a** The distribution of |*E*_*z*_|^2^ resembles the zero-order Bessel function *J*_0_. **b** The distribution of |*E*_*r*_|^2^ resembles the first-order Bessel function *J*_1_

For the case of the radially polarized incident light, as an example shown in Fig. 11, the intensity distribution in |*E*_*z*_|^2^ is identical to the zero-order Bessel function *J*_0_(*K*_*spp*_ ⋅ *n* ⋅ *r*), where *n* and *r* is the refractive index of the environmental medium and the radial distance to the optical axis, respectively. The FWHM of the focus is slightly larger than the size of the main lobe calculated with *J*_0_. Particularly, the simulations indicate that the non-diffraction beam can be realized in the quasi-far field. The SP waves, as a kind of evanescent wave, are decreased exponentially when propagating away from the exit surface, and the propagating distance in the vacuum can be calculated by [30]:

4 $$ {\delta}_d=\frac{1}{k_0}{\left|\frac{\mathrm{Re}\left({\varepsilon}_m\right)+{\varepsilon}_d}{{\varepsilon_d}^2}\right|}^{\frac{1}{2}} $$

where *ε*_d_ and *ε*_m_ are the permittivity of dielectric and metal, respectively. Thus, *δ*_d_ is 357 nm which is consistent with the simulation as shown in Fig. 8d. Therefore, the intensity of the SP waves at the focus can be neglected in the quasi-far-field region.

### II Shape of Focus

**Fig. 12:**
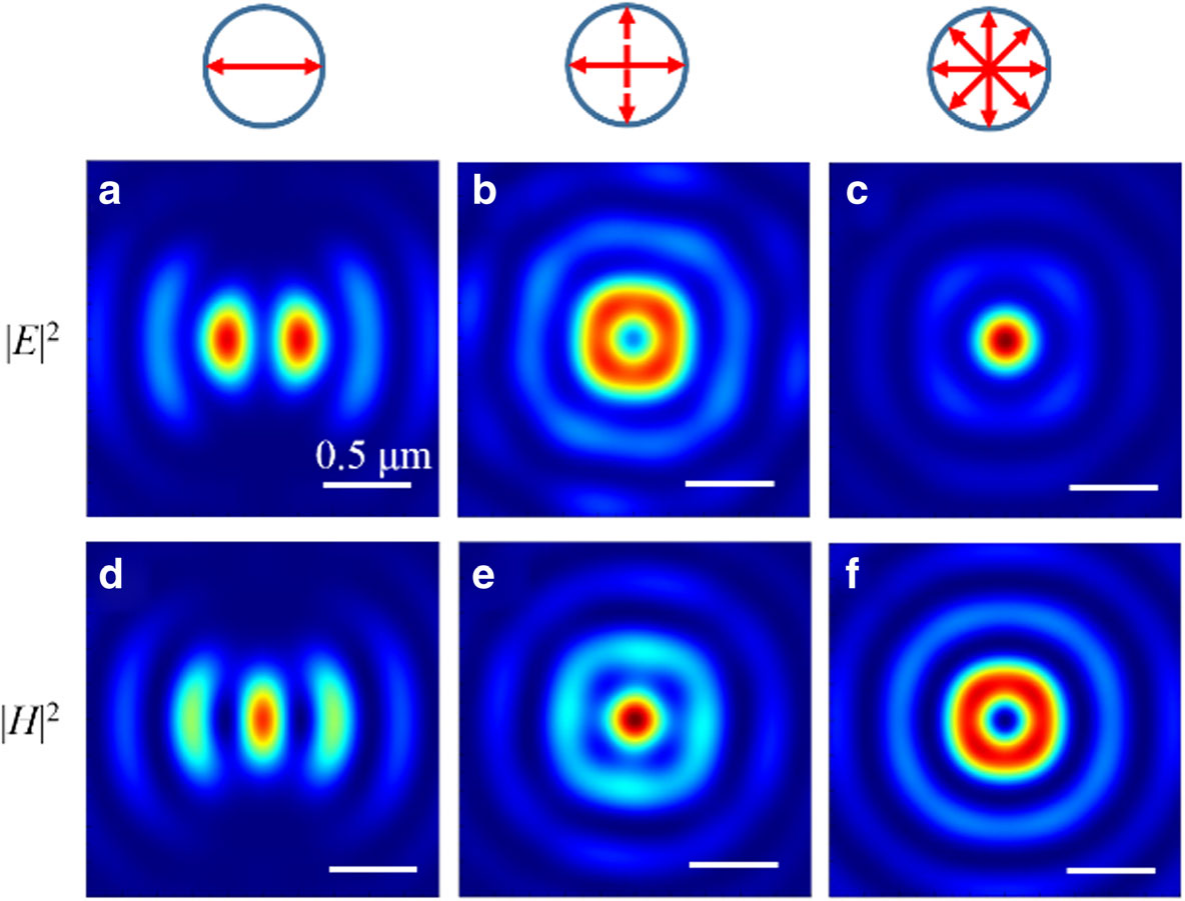
The normalized intensity patterns of the electric field |*E*|^2^ and magnetic field |*H*|^2^ in the focal plane when the NRPL is illuminated by the polarized light. **a** |*E*|^2^ and **d** |*H*|^2^ distribution with linearly polarized incident light. **b** |*E*|^2^ and **e** |*H*|^2^ distribution with circularly polarized incident light. **c** |*E*|^2^ and **f** |*H*|^2^ distribution with radially polarized incident light

By modulating the SOP, the elliptical-, circular-, and donut-shape foci can be realized in the focal plane, as presented in Fig. 12. The phenomenon is attributed to the subwavelength focal size, and we cannot realize the circular-type focus in both electric and magnetic fields at the same time. Thus, a donut-type focus is realized in the magnetic (or electric) fields, while a circular-type focus is achieved in the correspondingly electric (or magnetic) field. Particularly, because there is no longitudinal magnetic field, the intensity distribution of |*H*|^2^ is the same as the pattern of |*E*_*r*_|^2^. Furthermore, |*E*_*z*_|^2^ occupies about 80.0% of the total electric energy and the scale is not affected by the SOPs of the incident light.

### III Modulation of Focal Length

The simulated focal length in different cases is basically close to the desired position *f*_0_ (1300 nm), as shown in Table 1. But, we realize that the focal length in the transverse field |*E*_*r*_|^2^ is about 200 nm longer than that in the longitudinal field |*E*_*z*_|^2^, regardless of the SOP of the incident light and the deviation commonly exists.

**Table 1:** The focal length in the transverse and longitudinal electric field

	LP	CP	RP
|*E*_*r*_|^2^ (nm)	1425	1405	1455
|*E*_*z*_|^2^ (nm)	1215	1218	1275

*Note*: LP, CP, and RP represent for the linear polarization, circular polarization, and radial polarization of the incident light, respectively

**Fig. 13:**
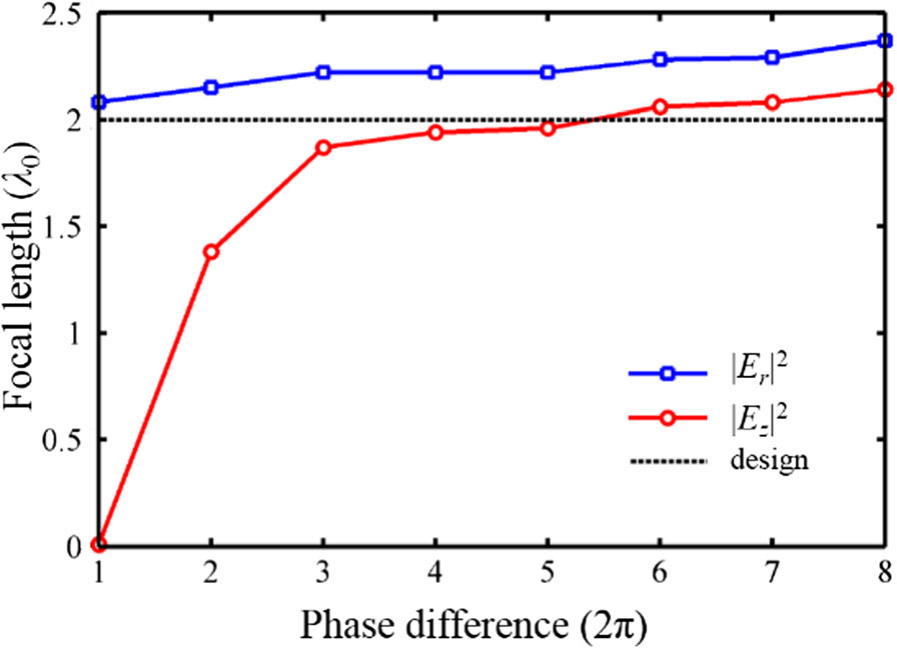
The focal length of the NRPL with the increase of total phase difference from 2π to 16π

In theory, the wavefront reconstruction theory is suitable for designing the NRPL with an arbitrary focal length from the near-field to the far-field region. However, whether the actual focal length of a designed plasmonic lens agrees well with the designed focal length depends on the total phase difference of the lens. The deviation may attribute to the distinction between the amplitude-type focus and phase-type focus [34]. Because the phase modulation in the MIM waveguide aims for the radial component, the focal length in |*E*_*r*_|^2^ can be modulated by the wavefront reconstruction theory, when the total phase difference of at least 2π is satisfied [15]. For the longitudinal component, a larger total phase difference (>10π) is advantageous to the consistency. As shown in Fig. 13, when the phase difference increases from 2π to 16π, correspondingly the NA of 0.75 to 0.96, the amplitude-type focus in |*E*_*z*_|^2^ moves from the output surface of the lens to the desired position. As the intensity distribution of |*E*|^2^ is decided by |*E*_*z*_|^2^, the NA can dramatically influence the focal length in the total electric field. However, the change of the focal length in |*E*_z_|^2^ decreases gradually, along with the increase of the total phase difference. On the other hand, the position of the phase-type focus in |*E*_*r*_|^2^ is relatively stable. When the NRPL with a high NA is applied, there is still a deviation in focal length derived based on the intensity distribution of |*E*_*x*_|^2^ and |*E*_*z*_|^2^, and the deviation almost keeps invariable. Therefore, the focal length of the NRPLs can be effectively controlled by the phase modulation and structural optimization, though the same focal length cannot be achieved in the transverse and longitudinal component fields.

### IV Focusing Performace in the Non-Coaxial Situation

**Fig. 14:**
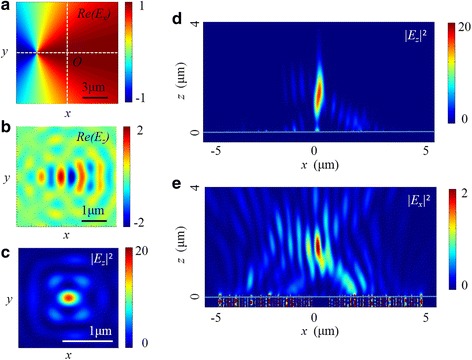
The intensity distribution of the NRPL in the non-coaxial situation. **a** The real part of *E*_*x*_ of radially polarized incident light. **b**, **c** The distribution of *Re(E*_*z*_ *)* and |*E*_*z*_|^2^ in the focal plane. **d**, **e** The distribution of |*E*_*z*_|^2^ and |*E*_*x*_|^2^ in the *x-z* plane

The non-coaxial situation is a common problem in the experiment, and its effect on the focusing performance should be considered. As shown in Fig. 14, the center of the radially polarized light deviates 3 μm from the optical axis of the NRPL along the *x* axis. Compared with Figs. 8 and 9, the intensity distributions both in *x-z* cross section and in focal plane are apparently changed. In the longitudinal electric field, an elliptical focus is located at 1340 nm away from the exit surface of the lens. The FWHMs in *x-z* and *y-z* planes are 0.51 and 0.38 *λ*_0_, respectively. On the other hand, the distribution in transverse field is also distorted, where the intensity of one side lobe is higher than the other one. Furthermore, compared with the coaxial condition, the decrease of the maximum intensity in the total electric field is more than 85%.

The preliminary simulation indicates that the non-coaxial situation indeed influences the intensity distribution and the desired focusing performance of lens. Therefore, it is essential to guarantee the coaxiality between the incident light and the lens center during the experiment.

## Conclusions

In summary, we build a NRPL with a high NA utilizing the wavefront reconstruction theory and the dispersion relation of the MIM waveguide. We also investigate the polarization-dependent focusing performance in the quasi-far field, including the focal length, FWHM, DOF, and the maximum intensity. The conventional polarized light, such as the linearly, circularly, radially, and azimuthally polarized light, are all considered. The simulations demonstrate the superfocusing capability of the designed NRPL. Utilizing the polarization-dependent property, the sub-diffraction-limit elliptical-, circular-, and donut-shape foci can be realized. However, one limitation of this work is that the proposed design strategy to realize the superfocusing performance of NRPLs is aimed for the quasi-far-field region, although to the best of our knowledge, the similar focusing capability in this region is rarely reported. In addition, we discover the underlying physical phenomenon on the focal shift and propose a more effective way to control the focusing position by employing both the transverse and longitudinal fields. There are considerable engineering applications for the nanoring-based superfocusing lenses, ranging from the super-resolution imaging, particle acceleration, quantum optical information processing to the optical data storage.

## Abbreviations

DOF: Depth of focus
FDTD: Finite-difference time-domain
FWHM: Full-width at half maximum
MIM: Metal-insulator-metal
NRPL: Nanoring-based plasmonic lenses
PML: Perfectly matched layer
SOP: State of polarization
SPPs: Surface plasmon polaritons
TE: Transverse electric
TM: Transverse magnetic
